# An Experimental Adult Zebrafish Model for *Shigella* Pathogenesis, Transmission, and Vaccine Efficacy Studies

**DOI:** 10.1128/spectrum.00347-22

**Published:** 2022-05-23

**Authors:** Debaki R. Howlader, Ushasi Bhaumik, Prolay Halder, Aishwarya Satpathy, Sounak Sarkar, Mrinalini Ghoshal, Suhrid Maiti, Jeffrey H. Withey, Jiro Mitobe, Shanta Dutta, Hemanta Koley

**Affiliations:** a Division of Bacteriology, ICMR-National Institute of Cholera and Enteric Diseases (ICMR-NICED), Kolkata, West Bengal, India; b Department of Biochemistry, Microbiology and Immunology, Wayne State University School of Medicine, Detroit, Michigan, USA; c Department of Bacteriology, National Institute of Infectious Diseases (NIID), Tokyo, Japan; University of North Dakota

**Keywords:** animal models, *Shigella*, shigellosis, zebrafish

## Abstract

Shigellosis has been a menace to society for ages. The absence of an effective vaccine against *Shigella*, improper sanitation, and unhygienic use of food and water allow the disease to flourish. *Shigella* can also be transmitted via natural water bodies. In the absence of a good animal model, the actual nature of pathogenesis and transmission remains unclear. Zebrafish larvae have previously been described as a model for *Shigella* pathogenesis. However, larval fish lack a mature intestinal microbiota and immune system. Here, the adult zebrafish was assessed as a potential model for *Shigella* pathogenesis. Their well-developed innate and adaptive immune responses mimic the mammalian immune system. *Shigella* showed a clear dose-, time-, and temperature-dependent colonization of the adult zebrafish gut. Efficacy of a three-dose immunization regime was tested using bath immunization with heat-killed trivalent *Shigella* immunogen. The present study demonstrates the efficacy of an adult zebrafish model for pathogenesis, transmission, and vaccine efficacy studies.

**IMPORTANCE** Shigellosis is a diarrheal disease that is prevalent in developing countries and especially dangerous in young children. Currently, animal models for shigellosis are unable to model some aspects of the infectious cycle. Here, we describe a new shigellosis model in adult zebrafish, an increasingly common model organism for studying bacterial pathogens. The zebrafish model can be used to study *Shigella* colonization, transmission, and immune responses, as well as test vaccine efficacy.

## INTRODUCTION

Shigellosis causes a significant economic and public health burden globally, resulting in ~164,000 mortalities annually (1). Symptoms of shigellosis range from mild watery diarrhea to inflammatory distress, including abdominal cramps along with blood- and/or mucus-containing stool. Immune-compromised individuals are at much higher risk due to the highly infective nature of the pathogen, with an infectious dose as low as 10 bacteria (2). *Shigella* transmits primarily through water; hence, countries with underdeveloped sanitary systems are at heightened risk for infection. Mice are generally resistant to oral delivery of *Shigella*. However, the disease can be modeled in mice via surgical and/or immunological manipulations (3–9). In a guinea pig luminal model, the appropriate symptoms occur, but this model is difficult to execute and needs highly trained laboratory personnel (9). Other models require prior antibiotic administration and opium or ketamine treatment, making them unsuitable for studies of natural disease biology (10). None of these models address the transmissibility of the pathogen via water.

Zebrafish have an immune system much like that of mammals that includes innate and adaptive responses. Their small size, ease of maintenance, and relatively low cost make them an ideal animal model (11, 12). Previous studies have used larval zebrafish as a model for *Shigella* infection (12). However, larvae lack an adaptive immune response and have underdeveloped intestinal microbiota, and transmission studies are problematic. Adult fish, on the other hand, have a mature adaptive immune response and fully developed microbiota for pathogenesis studies.

In this study, a new experimental zebrafish model was developed. It was used to assess colonization and pathogenesis as well as to study protective efficacy of a newly developed heat-killed immunogen. *Shigella* depends largely on its type three secretion system (TTSS) for pathogenesis. At 37°C, a tip complex (TC) is formed by IpaB and IpaD (13), and the chaperone Hfq stabilizes the secretion of TTSS. To facilitate this process in the new model, fish were kept at 37°C, instead of their usual 28°C habitat in the laboratory. We have found that *Shigella* can infect wild-type adult fish at 37°C and transmit the infection to naive fish. Reducing the housing temperature to 30°C hampered the infection. We have also evaluated the usefulness of this model in a vaccine efficacy study. This model can thus be used for pathogenesis studies of *Shigella* in waterbodies. This study also covers the lacunae of transmission of *Shigella* from waterbodies into humans.

## RESULTS

### *Shigella* can colonize the adult zebrafish intestine, resulting in pathogenesis.

**FIG 1:**
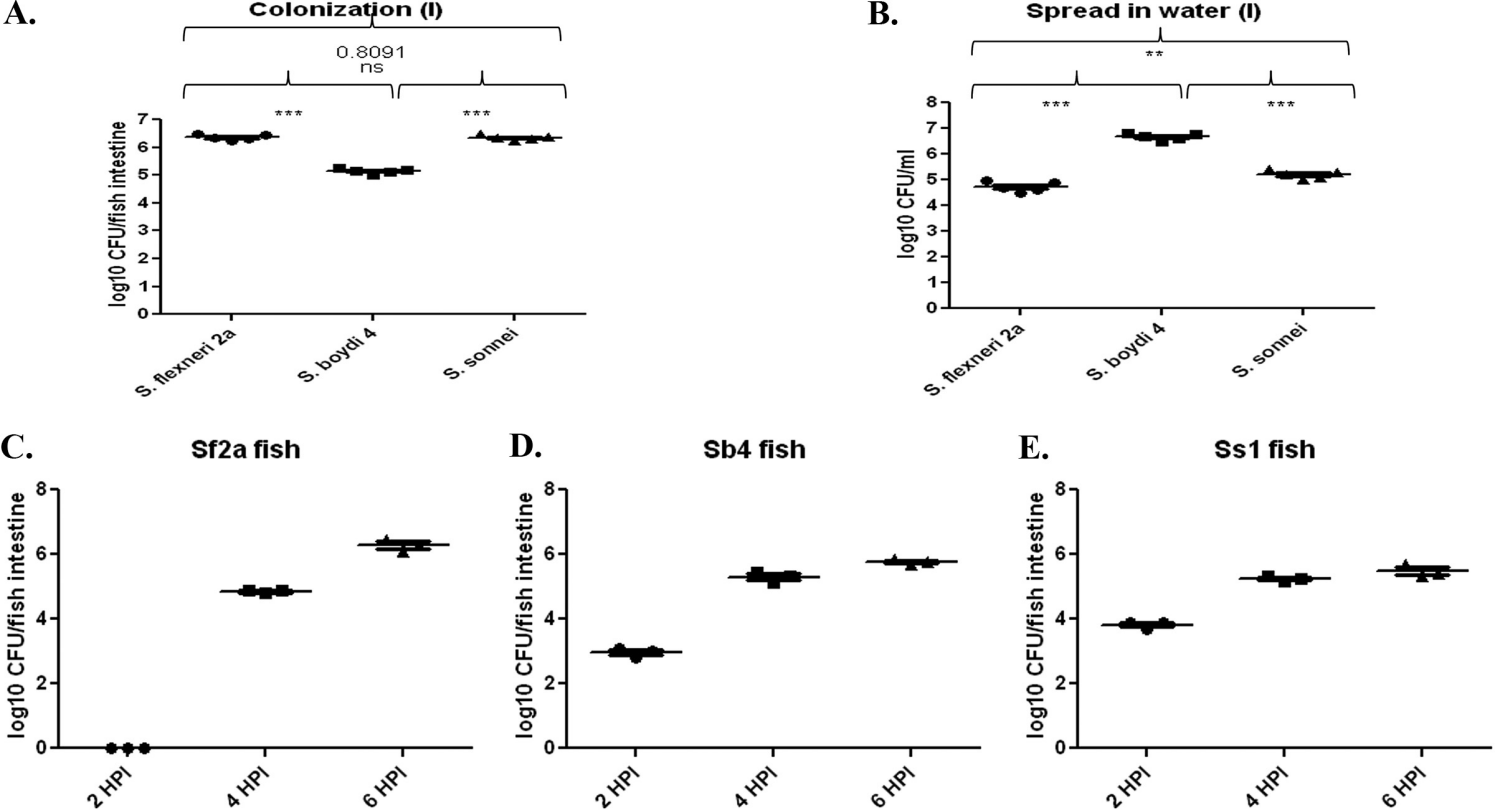
*Shigella* spp. colonize zebrafish intestine. Naive fish were challenged with 1 × 10^6^ CFU/mL of respective *Shigella* sp. for 24 h. Water was changed at 3 hpi, then at 21 hpi. (A) Colonization of *Shigella* sp. in fish gut. (B) Expulsion of *Shigella* in fish water. (C to E) Time-dependent infection of zebrafish by (C) S. flexneri *2a*, (D) S. boydii *4*, and (E) S. sonnei phase I. The horizontal bar represents the mean bacterial load. Tukey’s multiple-comparison test was used to determine the statistical significance between the groups. Each dot represents the result from one fish. *n* = 5 for A and B, *n* = 3 for C, D, and E. *, *P* < 0.05; ***, *P* < 0.001.

At 30°C, *Shigella* was unable to cause pathogenesis despite having high CFU in water. However, fish kept at 37°C for 24 h were significantly colonized by *Shigella* (Fig. 1). Fish were inoculated with 1 × 10^6^ CFU/mL. Typically,10^5^ to 10^7^ CFU/fish intestine was found to be recovered after 24 h of incubation at 37°C (Fig. 1A). A total of 10^5^ to 10^7^ CFU/mL of bacteria was recovered from the water, suggesting abundant *Shigella* replication in the fish intestines followed by excretion into the water (Fig. 1B). Among the three strains tested, *Sb4* had the lowest bacterial load in the fish intestine. However, fish infected with *Sb4* excreted a higher number of bacteria into the water for reasons that are currently unclear. Since the water was changed at 3 hours postinfection (hpi) and then subsequently at 21 hpi, repeated recovery of the same bacteria confirms continued bacterial replication in the fish gut. Time point analysis for colonization of fish indicates that the bacteria were able to colonize the fish intestine as soon as 2 hpi (Fig. 1C to E), except *Sf2a*. However, at later time points, comparable numbers of bacteria were observed for all three strains. Time dependence was also observed with increasing bacterial load at later time points (Fig. 1C to E). Subsequently, 50% lethal dose (LD_50_) was determined to be 2 × 10^7^ CFU/mL for all the strains (Supplemental File 1). Higher doses resulted in killing more than half (>50%) of the fish population, whereas lower doses resulted in <50% fish death. A divergence in colonization and survival of bacteria was seen at 30°C following an LD_50_ infection (Fig. S2A to C). *P* values are shown in Table 1.

**TABLE 1:** Statistical analysis of hour-dependent colonization in fish gut^*a*^

Group	Statistical significance in:		
	Sf2a gut	Sb4 gut	SsI gut
2 hpi vs 4 hpi	***	***	***
2 hpi vs 6 hpi	***	***	***
4 hpi vs 6 hpi	***	*	ns

a Hour-dependent colonization in fish gut. Statistical significance was tested using Tukey’s multiple-comparison test. *, *P* < 0.05; ***, *P* < 0.001; ns, not significant.

### Various degrees of infection were found in fish over a day-dependent colonization study.

**FIG 2:**
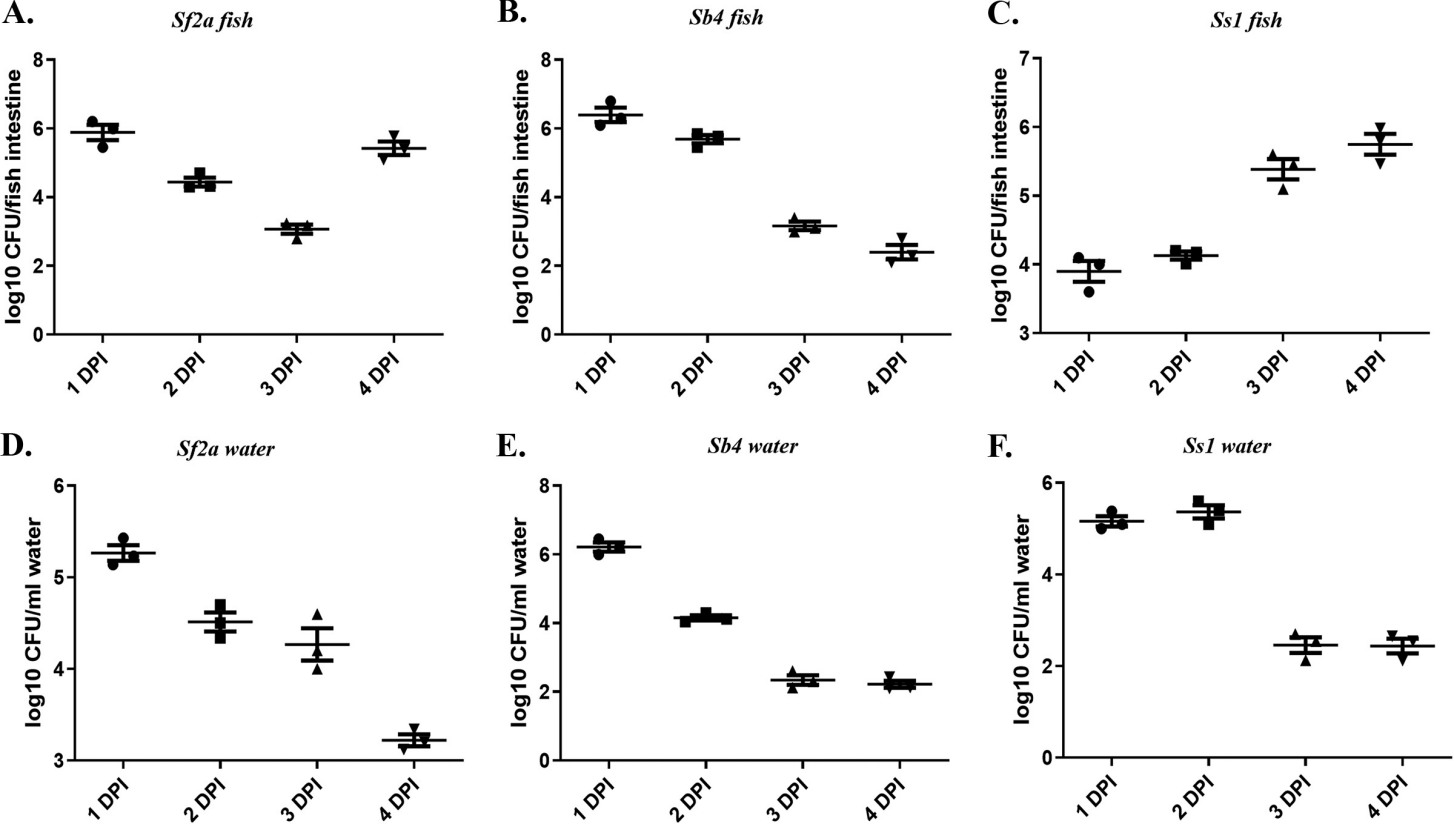
Day-dependent colonization study in naive fish. Naive fish were challenged with 1 × 10^6^ CFU/mL bacteria and kept for 4 days. Water was changed at 3 hpi, then at 21 hpi, and then every subsequent 24 h. Colonization of (A) S. flexneri *2a*, (B) S. boydii *4*, and (C) S. sonnei phase I in fish gut is shown. Presence of (D) S. flexneri *2a*, (E) S. boydii *4* and (F) S. sonnei phase I in water were shown as well. The horizontal bar represents the mean bacterial load. Tukey’s multiple-comparison test was used to determine the statistical significance between the groups. Each dot represents the result from one fish. *n* = 3; *, *P* < 0.05; **, *P* < 0.005; ***, *P* < 0.001.

As already stated, the infection was detectable even at 2 hpi and was found to remain at a detectable level at least until 96 hpi. A gradual drop in CFU was observed for *Sf2a* over a period of up to 3 days postinfection (dpi), but then CFU increased at 4 dpi (Fig. 2A). A time-dependent reduction of bacteria in water was also observed (Fig. 2D). In the case of *Sb4*, a sustained presence was observed for 2 days, but then a rapid decline was observed (Fig. 2B). The observed 10^4^ to 10^6^ CFU/mL in the water was likely due to high infection and internalization of the bacteria in fish intestine followed by rapid discharge (Fig. 2E). In the case of *SsI*, a time-dependent increase in colonization was seen with 10^4^ to 10^6^ CFU/fish intestine over the period (Fig. 2C). Excreted bacteria in water were found to be around 10^6^ CFU/mL but soon decreased to less than 10^3^ CFU/mL at 3 dpi and declined until day 4 (Fig. 2F). *P* values are shown in Table 2.

**TABLE 2:** Statistical analysis of day-dependent colonization in fish gut and expulsion in water^*a*^

Group	Statistical significance in:					
	Sf2a gut	Sb4 gut	SsI gut	Sf2a water	Sb4 water	SsI water
1 dpi vs 2 dpi	**	ns	ns	**	***	ns
1 dpi vs 3 dpi	***	***	***	**	***	***
1 dpi vs 4 dpi	ns	***	***	***	***	***
2 dpi vs 3 dpi	**	***	**	ns	***	***
2 dpi vs 4 dpi	*	***	***	***	***	***
3 dpi vs 4 dpi	***	*	ns	**	ns	ns

a Day-dependent colonization in fish gut and expulsion in water. Statistical significance was tested using Tukey’s multiple-comparison test. *, *P* < 0.05; **, *P* < 0.005; ***, *P* < 0.001; ns, not significant.

**FIG 3:**
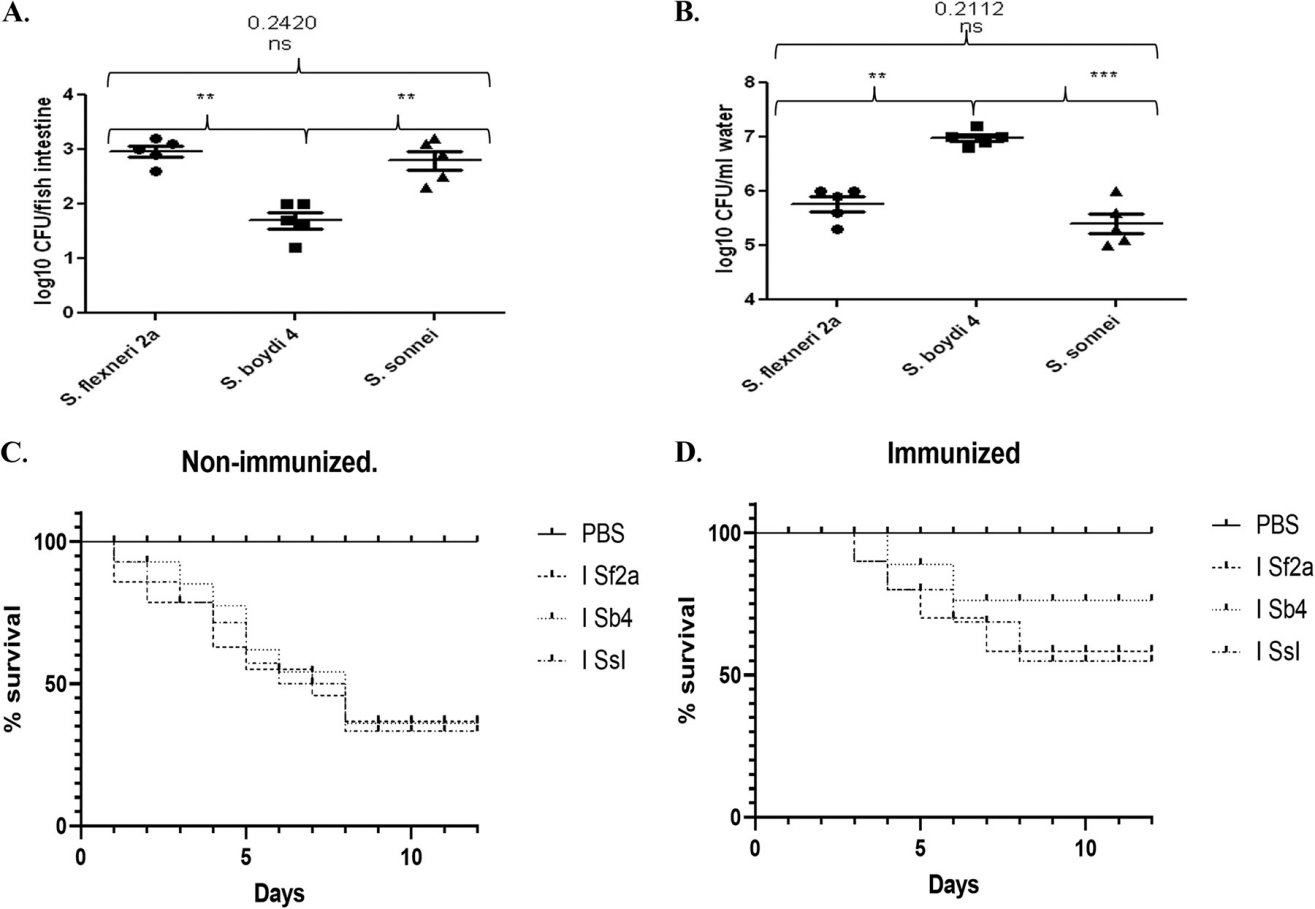
Protective efficacy studies. Fish were immunized with a trivalent heat-killed immunogen in a prime-boost-boost manner. At the 35th day, they were challenged with LD_50_ of *Shigella*. (A) Protective efficacy. (B) Expulsion of bacteria into water. The horizontal bar represents the mean bacterial load. Each dot in A and B represents the result from one fish. For survival study, 20 zebrafish per group were challenged with LD_50_ of each challenge bacteria and kept for 12 days. In every case, water was changed once at 3 hpi, then at 21 hpi, and then every subsequent 24 h. (C) Survival of naive fish. (D) Survival of immunized fish. Survival curves were analyzed by log-rank (Mantle-Cox) test. They were also analyzed by the Gehan-Breslow-Wilcoxon test for early time point death analysis. Significant difference was observed between infected nonimmunized groups versus PBS, while no significant differences were found between immunized groups and PBS. *, *P* < 0.05; **, *P* < 0.005; ***, *P* < 0.001.

**Trivalent heat-killed *Shigella* immunogen blocks *Shigella* colonization in zebrafish gut.** Previous work in mouse models found that a trivalent heat-killed *Shigella* immunogen could induce a protective immune response. To determine whether the immunogen would also induce protection in the new zebrafish model, fish were bath immunized with 3 × 10^9^ CFU/mL heat-killed bacteria on days 0, 14, and 28 and then challenged with live bacteria. Eighteen hours following challenge with one LD_50_, intestines were removed and assessed for bacterial burden. Intestines isolated from the immunized fish contained fewer bacteria than intestines from the nonimmunized fish. Nearly 10^3^ CFU were recovered per intestine for *Sf2a* and *SsI*, whereas 10^2^ CFU/intestine were recovered from *Sb4*-challenged immunized fish (Fig. 3A).

In the initial colonization study, naive fish were infected with 1 × 10^6^ CFU/mL of bacteria, resulting in a high bacteria burden in the fish (Fig. 1A). The water in beakers containing nonimmunized fish (Fig. 1B) had a number of bacteria comparable to that of the water in beakers containing immunized fish (Fig. 3B). However, the immunized fish had fewer bacteria in their gut. This was thought to be a direct effect of immunization, where immunized fish successfully killed the inoculated bacteria (Fig. S3) and hindered the remainder from attaching to the gut wall, leading to their expulsion. Immunization was also found to be protective against invasive infection by *Shigella*. The bacteria were observed to invade internal organs such as heart and liver of nonimmunized fish. Presence of *Shigella* in the heart indicates systemic spread of the bacteria in the host. Much less colonization was observed in the internal organs of immunized fish (Fig. S4A and B).

**Immunization improved zebrafish survival against long-term *Shigella* infection.** Both immunized and nonimmunized fish were challenged with an LD_50_ of the respective *Shigella* strain. Fish death began to occur at 1 dpi in nonimmunized fish and at 3 dpi in immunized fish. The overall survival of immunized fish was significantly higher than that of the nonimmunized fish (Fig. 3C and D). At the end of 12 days, 55 to 60% of the nonimmunized fish had survived the infection, whereas 85 to 90% of the immunized fish had survived the infection. This suggests that the immunogen generated a protective response, resulting in improved fish survival.

### Effects of immunization on transmission of *Shigella* from infected to naive fish.

**FIG 4:**
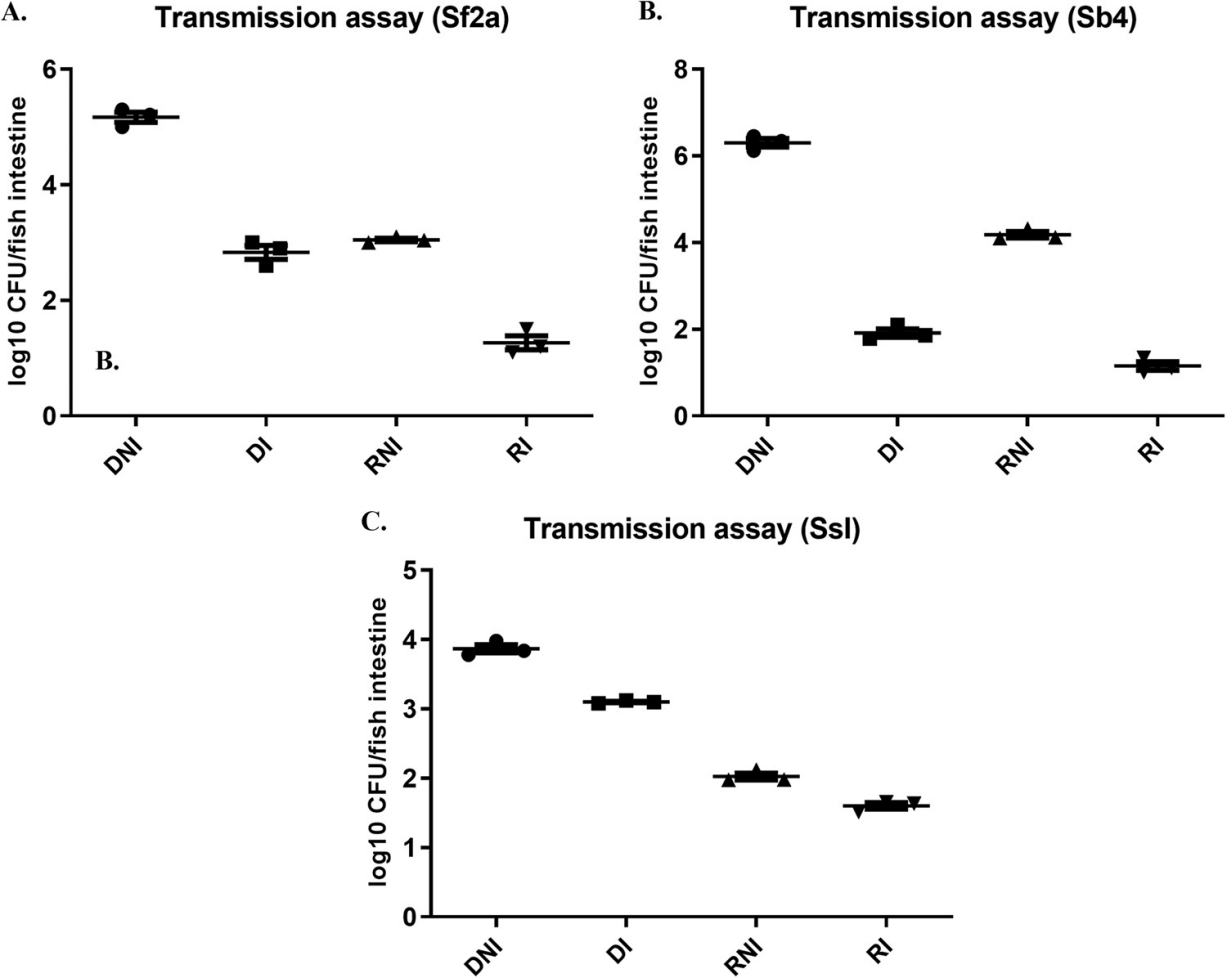
*Shigella* transmission in immunized versus nonimmunized fish. Three fish per group were exposed to each of the indicated bacteria and observed for 3 h. At 3 h, the water was changed, and 3 naive fish were transferred into the beaker. The fish were observed until 18 hpi. Bacterial load was determined for (A) S. flexneri *2a*, (B) S. boydii *4* and (C) S. sonnei phase I. The horizontal bar represents the mean bacterial load. Each dot represents the result from one fish. DNI, donor-nonimmunized fish; RNI, recipient-nonimmunized fish, DI, donor-immunized fish; RI, recipient-immunized fish. Tukey’s multiple-comparison test was used to determine the statistical significance between the groups. **, *P* < 0.005; ***, *P* < 0.001.

Bacterial passage from infected to uninfected fish relies on the efficient transmissibility of the bacterium. Blocking this would help reduce the spread. Four groups of fish were used in the transmission experiment, i.e., donor-nonimmunized (DNI), recipient-nonimmunized (RNI), donor-immunized (DI), and recipient-immunized (RI) fish. All DNI groups showed the usual high CFU counts (10^4^ to 10^6^ CFU/fish intestine) after an exposure with one LD_50_ of *Shigella*. Recipient-nonimmunized fish were found to have 10^2^ to 10^4^ CFU/fish intestine, which illustrates that this model system can be used to assess transmission. DI groups, on the other hand, showed much less colonization, around 10^2^ to 10^3^ CFU/fish intestine, as expected based on the results described above that indicated that immunization lowers bacterial burden. This result reiterates the protective efficacy of the trivalent immunogen. Interestingly, the RI groups had lower numbers of intestinal *Shigella*, in the range of only 10^1^ to 10^2^ CFU/fish intestine. This result suggests that the trivalent immunogen can also restrict transmission to some extent but fails to prevent it completely (Fig. 4A to C). Marked differences were observed between these four groups in the cases of *Sf2a* and *Sb4*. *SsI* had little difference in bacterial load in the RNI and RI groups. It is thus concluded that the trivalent immunogen confers differing levels of protection against different *Shigella* species. The statistical significance is shown in Table 3.

**TABLE 3:** Statistical analysis of the transmission experiment^*a*^

Group	Statistical significance in:		
	Sf2a gut	Sb4 gut	SsI gut
DNI vs DI	***	***	***
DNI vs RNI	***	***	***
DNI vs RI	***	***	***
DI vs RNI	ns	***	***
DI vs RI	***	**	***
RNI vs RI	***	***	***

a Transmission of *Shigella* from infected to uninfected fish was observed. Statistical significance was tested using Tukey’s multiple-comparison test. **, *P* < 0.005; ***, *P* < 0.001; ns, not significant.

### Colonization depends on O-antigen structure as well as the type 3 secretion system.

**FIG 5:**
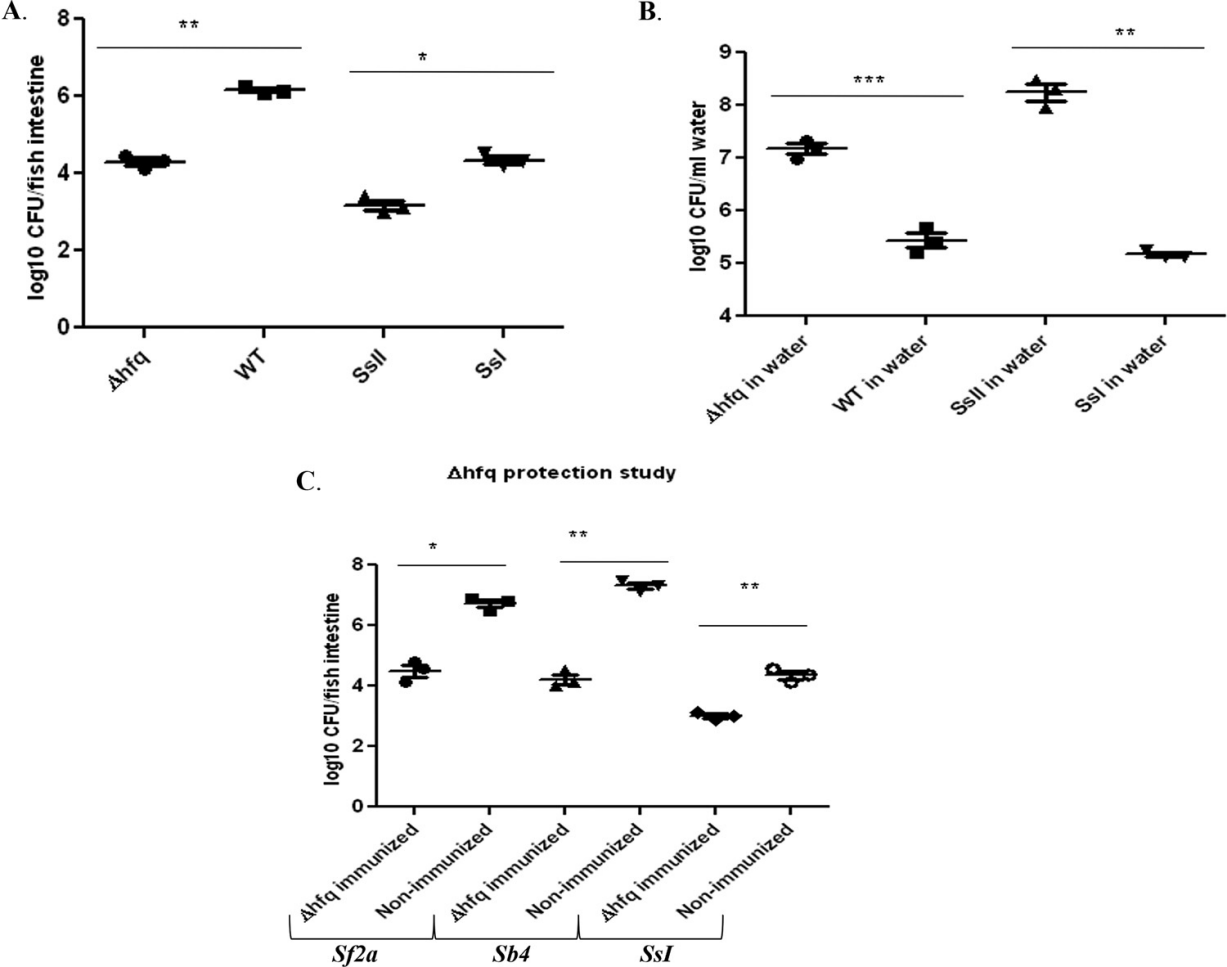
Comparative analysis of colonization between Δ*hfq* and wild-type *Shigella* strains, and protective efficacy of the live-attenuated Δ*hfq* strain. (A) Fish were colonized with Shigella flexneri *2a* Δ*hfq*, wild-type (WT) Shigella flexneri *2a*, Shigella sonnei phase II, and Shigella sonnei phase I. (B) Presence of these same bacteria in the water following colonization. (C) Δ*hfq*-immunized fish were challenged with LD_50_ of Shigella flexneri *2a*, Shigella boydii *4*, and Shigella sonnei phase I wild-type strains. Colonization was compared with that of the nonimmunized fish. Each dot represents the result from one fish. *n* = 3; *, *P* < 0.05; **, *P* < 0.005; ***, *P* < 0.001.

Hfq is an important TTSS regulator and RNA-binding protein/chaperone. *hfq* deletion causes constitutive production and secretion of TTSS effectors (14). It is hypothesized that TTSS plays an important role in the colonization of animals. To assess whether a TTSS defect causes colonization differences, we checked the level of colonization of a Δ*hfq* Shigella flexneri strain in fish intestine. Δ*hfq* S. flexneri colonization was found to be in the range of 10^4^ to 10^5^ CFU/fish gut, and Shigella sonnei phase II colonization was in the range of 10^3^ to 10^4^ CFU/fish gut (Fig. 5A). Since *SsI* and *SsII* are different in respect to both the secretion apparatus and O antigen, our result suggests that not only the TTSS proteins but also the structure of O antigen might be important for colonization. However, it is not possible to clarify further within the scope of this work. There could be an additional impact of the type six secretion system (T6SS) in *SsI* but not in *Sf2a* or *SsII* (15); however, we did not assess this in the present study. Wild-type *Sf2a* and *SsI* showed high colonization, as expected (Fig. 5A). Excretion in water was also assessed, and both Δ*hfq* and *SsII* excreted more bacteria than their wild-type counterparts (Fig. 5B). The Δ*hfq* S. flexneri strain was later used as a live-attenuated immunogen. The Δ*hfq* S. flexneri immunized fish were protected from *Sf2a*, *Sb4*, and *SsI*. Fewer bacteria were recovered from immunized fish guts than from their nonimmunized counterparts (Fig. 5C).

### Zebrafish as a model organism for testing therapeutics.

**FIG 6:**
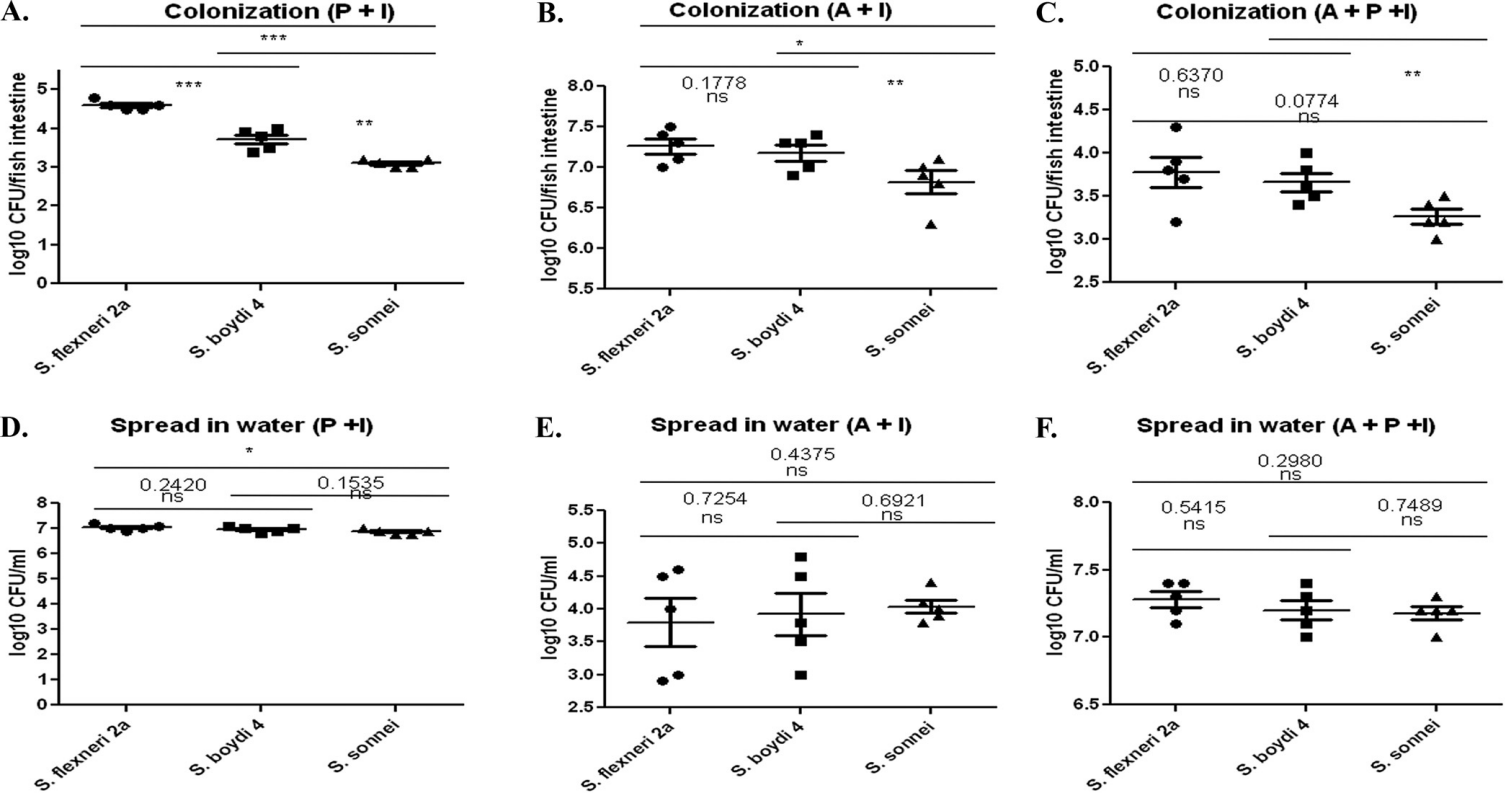
Testing a probiotic/commensal mixture as a potential therapeutic in zebrafish. The fish were (A) treated with probiotic/commensal mixture and then infected with *Shigella* sp. challenge strains (P+I), (B) treated with an antibiotic cocktail to damage gut microbiota before infection with *Shigella* sp. (A+I), or (C) treated with antibiotics, then treated with probiotic/commensal mixture, and then infected with *Shigella* sp. (A+P+I). Bacterial expulsion in water was also assessed and is shown in panels D, E, and F, respectively. Each dot represents the result from one fish. The horizontal bar represents the mean bacterial load. *n* = 5; *, *P* < 0.05; **, *P* < 0.005; ***, *P* < 0.001. P, probiotic treated; A, antibiotic treated; I, infected with bacteria.

Use of zebrafish as a therapeutic model organism was assessed by treating the fish with antibiotics, a probiotic/commensal mixture, and/or both prior to *Shigella* challenge. Treatment with a probiotic/commensal mixture provides a physical barrier that inhibits *Shigella* from attaching itself to the gut floor. Antibiotic treatment reduces the natural microbiota, and thus, this group of fish experienced the highest colonization among all groups tested. The last group, in which fish were treated with antibiotics followed by the probiotic/commensal mixture, experienced the lowest *Shigella* colonization (Fig. 6A to C). Bacterial expulsion into water was higher in groups one and three and lower in group two (Fig. 6D to F). A marked difference can be seen in the hematoxylin and eosin (H&E)-stained intestines between infected and uninfected control fish (Fig. S5). Antibiotic treatment to reduce the natural gut microbiota caused a massive tissue infiltration by the bacteria (Fig. S5C), whereas this was absent in probiotic-treated, *Shigella*-infected fish gut (Fig. S5D).

## DISCUSSION

Water is an essential factor for the existence of life. Inadequate sanitary systems, along with unhygienic usage of water, leads to various waterborne acute microbial diarrheal diseases (16, 17). Both developing and developed countries are at a risk of infection through waterborne diseases. Approximately 477,000 people suffer from severe waterborne disease every year in the United States (18).

Since its discovery in 1897 (19), pathogenesis of *Shigella* has been tested in various animal models and using *in vitro* and *ex vivo* settings. The bacteria transmit through the fecal-oral route and can survive in water at room temperature up to 6 months (20, 21). Although its transmission via solid food and other environmental means has been shown in the literature, the mode of spread in natural water bodies has not yet been evaluated in detail. *Shigella* is also one of the 12 priority pathogens listed by the WHO that requires urgent action (22). Here, we have developed an experimental adult zebrafish model to replicate the aquatic environmental situation. We further assessed the protective efficacy of a trivalent *Shigella* heat-killed immunogen using this model. The potential of this model to be used to assess therapeutics was also evaluated.

None of the animal models for *Shigella* pathogenesis (3–9) can assess the ecological spread of the organism. Some of the models are specific for a single *Shigella* sp., and infection from other *Shigella* spp. cannot be assessed using that model. Moreover, trained personnel are a prerequisite to execute the models effectively. For instance, the surgical rabbit model for *Sf2a* needs the pathogen to be administered with precision into the colon following a cecal bypass. This model is effective, but it is unsuitable to use as a natural host model (3). The piglet model for Shigella dysenteriae 1 (*Sd1*) is a gnotobiotic animal model and, hence, is not a natural model of infection (4). The rhesus monkey model for *Sd1* is expensive and troublesome to execute due to animal ethical considerations (5). Guinea pig models of *Shigella* also have limitations that keep them from being usable as natural infection models (8, 9). The suckling mouse model for *Sf5a* is another artificial model in an animal that lacks a mature immune response and significant intestinal microbiota (6). The intraperitoneal mouse infection model is being heavily used presently due to its reproducibility and ease of handling (7). However, it is not a natural infection model but an induced one. Last, an NAIP-NLRC4 oral infection model for *Shigella* has recently been described (23). This model can be used in transmission experiments as a fecal-oral transmission model.

It is evident from the literature that an animal model is needed to study *Shigella* transmission in the environment. Reduction in transmission would reduce the spread of the disease. Zebrafish are an excellent model organism due to their ease of maintenance, reproducibility of data, and anatomical and immunological aspects that are generally similar to those of human (12). Zebrafish larvae have been used as a model for bacterial phagocytosis and autophagy due to their optical transparency, which helps in noninvasive real-time *in vivo* imaging techniques (24). Although several studies have been carried out using zebrafish larvae, in the context of *Shigella* sp. infection, the larvae (12, 25) cannot be used for transmission assays. Moreover, mature intestinal microbiota and an adaptive immune system are absent in the larvae, limiting their use. Since adult fish are susceptible to *Enterobacteriaceae* (both *Shigella* and *Salmonella*) (26), it is worth trying to develop a model to study the pathogenesis and spread of these organisms. Spread of these diseases will naturally be high in low-income countries with less-developed sanitary systems and unhygienic use of water.

Dosage greater than 10^8^ CFU/mL killed the adult fish within a short time. In a time course analysis, the infection ensued at 2 hpi, except for *Sf2a*, and it later reached 10^6^ CFU/fish intestine for all *Shigella* strains tested. The observed difference in infection between *Shigella* species might be a result of variability in the kinetics of the infection. Intriguing findings were noted while comparing the infection in fish and excretion into water. In almost all cases, the levels measured in water were found to be inversely proportional to the levels in the fish gut. This is notably different from the Vibrio cholerae adult zebrafish model, in which excreted bacteria in water paralleled the levels in fish intestine (27). It is worth noting the infection curve of *SsI*, where the bacterial load rose over time, which was opposite for other *Shigella* species. An inconsistency between *Shigella s*trains was also observed in colonization experiments at 24 h. *Sf2a* and *SsI* were found to be in the range of 6 to 7 log_10_, whereas *Sb4* was only around 5 log_10_. This inconsistency might have been a result of fish physiology. Since they were kept at 37°C, which is not the optimum physiological temperature, the fish might have reacted differently to different *Shigella* strains, but the reasons remain unclear at present. For *Sf2a*, infection at 4 dpi showed a significant increase over that at 3 dpi. This is an intriguing observation, but the mechanism for this is beyond the scope of this initial study.

This study also provides new evidence about the infection pattern of *SsI*. Being the main shigellosis-causing strain in developed countries (15), it may link transmission of the bacteria via different water bodies. The huge amount of ballast water in cargo ships from different parts of the world may provide the inoculum. There is evidence that *Shigella* passaged strains from fish gut are more infectious in nature (26). Therefore, infection from *Shigella* and its transmission via fish in water may act as a double-edged sword.

Infections from intracellular pathogens in aquaculture and in natural water bodies have increased in the recent past, and the need for an effective vaccine has become profoundly apparent (16, 17). Immune-stimulatory effects of heat-killed bacteria were evaluated in various previous studies (11, 28). Heat-killed Enterococcus faecalis activates cell-mediated immunity in fish, which is essential against intracellular pathogens (29). Effectiveness and usefulness of heat-killed bacteria over those of other immunogens have been assessed and often found to be more favorable (30–32). Although live-attenuated bacteria provide a better immune-stimulatory effect, there is an added risk of reversion into the virulent wild-type form (33). Use of several acellular vaccines also has limitations, since they are more prone to degradation, and some are expensive to produce (34), limiting their usage in low- and middle-income countries (LMIC).

This study found that using a three-dose regimen of heat-killed *Shigella* provided significant protection to the fish and limited the transmission of *Shigella* in water. Systemic invasive infection was also found to be reduced in immunized fish. Based on the epidemiological evidence, selection of the strains was important in the study, and finding satisfactory protection against these three prevalent strains supports the relevance of the work.

Pathogenesis is a complex phenomenon that decides the fate of an organism. Other than its plasmid-borne effector proteins (35), *Shigella* relies on its O-antigen structure to cause host damage (36). The TTSS is a needle-like complex that transfers effector proteins into the host intestinal epithelium and initiates the infection. *Sf2a* Δ*hfq* lacks the RNA chaperone Hfq and has constitutive TTSS activity. *hfq* suppression also represses *rpoE* and *rpoS* response regulators, which are important during stress conditions and in the stationary phase of bacterial growth. Δ*hfq* causes massive upregulation of TTSS effector secretion and thus would theoretically damage its host more than the wild type does. In our experiments, secretion of TTSS effectors does not have sole control over *Shigella* virulence, since the Δ*hfq* strain still cannot cause massive damage in the fish. Shigella sonnei phase II has a different O-antigen profile (37), and we observed a 2- to 3-fold colonization decrease compared to that of the pathogenic *SsI*. Δ*hfq* mutants have been used successfully in previous studies as an immunogen that confers cross-protection (14). Here, we observed moderate cross-protection against *Sf2a*, *Sb4*, and *SsI*. The observed difference in *SsI* could have been a result of other factors. Evolutionarily, *SsI* is different from *Sf2a* and *Sb4*. At the molecular level, *SsI* has an extra secretion system, namely, T6SS, whose genes are sparsely present in other *Shigella* strains. Increased fitness for interbacterial competition and niche occupancy may enhance fitness and lead to prolonged infection.

Fish blood contains a complement system just as mammalian blood does. These complement factors are heat-labile and contain the same features as their mammalian counterparts, i.e., ability to lyse bacteria via classical pathway, among others (37, 38). Here, immunized fish heart extract caused lysis of wild-type bacteria, and as a result, fewer bacteria were recovered. These data suggest the involvement of the fish immune system to reduce the bacterial load via complement and immunoglobulins specific against *Shigella*. In-depth studies on fish immunology were beyond the scope of this preliminary study.

The adult zebrafish was also tested for its potential use as a therapeutic model. As a proof of concept, it was hypothesized that antibiotic treatment will increase *Shigella* colonization, whereas probiotic/commensal treatment will help reduce and/or inhibit *Shigella* colonization in the fish gut. Antibiotic treatment significantly reduces the intestinal microbiota, and thus, *Shigella* can easily take up the available niche and exceed its “normal” colonization ability, as seen earlier. As a result of bacterial internalization, fewer bacteria were expelled into the water. On the other hand, probiotic/commensal treatment with or without prior antibiotic treatment reduced or inhibited the infection.

In summary, this new experimental model should be helpful for studies on *Shigella* pathogenesis, immune responses, and therapeutics.

## MATERIALS AND METHODS

### Bacterial strains and culture conditions.

Shigella flexneri *2a* 2457T (*Sf2a*), Shigella boydii *4* BCH 612 (*Sb4*), Shigella sonnei phase I IDH 00968 (*SsI*), and Shigella sonnei phase II IDH 00968 (*SsII*) were obtained from ICMR-National Institute of Cholera and Enteric Diseases (ICMR-NICED). Shigella flexneri *2a* Δ*hfq* (*Sf2a* Δ*hfq*) was constructed separately by Jiro Mitobe. All strains were preserved at −80°C in 8% glycerol containing brain heart infusion broth (BHIB; BD Difco). The bacteria were grown on tryptic soy agar (TSA; BD Difco) and tryptic soy broth (TSB; BD Difco) at 37°C. The Δ*hfq* strain was grown on LB Lennox (BD Difco) medium under appropriate antibiotic pressure.

### Zebrafish.

Adult wild-type zebrafish were maintained in previously stated conditions (11). All fish were 6 to 9 months old and belonged to the same stock with the same genetic background. Postinfection, fish were kept at either 30°C or 37°C, as needed. In all other cases, fish were kept at 28°C. During water changes, fish were moved to a different tank and the previous tank was rinsed with hot water (>80°C) and then dried with cotton cloths to ensure removal of any remaining water and/or bacteria. Fish were marked on dorsal fin for identification. All the animal experiments were approved by the Animal Ethical committee of ICMR-NICED: PRO/117/June 2015 to June 2018.

### Ethical statement.

Fish were monitored every day at 8-h intervals. Any behavioral changes, such as jumping from the tank, slow or fast movement, gulping for air, or moving over the base of the beaker, were noted and considered a potential sign of morbidity. No clinical signs of morbidity were reported during the entire study period. Microbiological assessments of fish intestine and aquarium water were done twice weekly. Fish were kept at 30°C and 37°C, wherever needed. Colonization and survival assays resulted in sudden fish death rather than pronounced clinical signs of morbidity. No humane endpoint analysis was possible.

### Colonization assay.

As a proof of concept, initial colonization and time-dependent studies were carried out by inoculation of 1 × 10^6^ CFU/mL of each bacterium separately into 200 mL water. The dosage was determined *a posteriori* and also depended on previous work in the field (24). At designated time points, fish were euthanized, thoroughly cleaned in phosphate-buffered saline (PBS) plus gentamicin (50 μg/mL, Gibco, 10 min/wash), and dissected. Intestine and other internal organs were merged in PBS plus gentamicin followed by two further washes in PBS only to remove excess gentamicin and homogenized in 0.1% Triton X-100 (catalog no. T6878, Sigma, USA)-containing PBS. Homogenates were serially diluted and plated. Presence of replicating bacteria in water was assessed, as well. Dependence on the regulation of TTSS was checked with a mutant strain of *Sf2a*, which is unable to produce the chaperone *hfq*. Dependence on O antigen and other virulence factors for colonization was checked via *SsI* and *SsII* strains. In every case, the number of fish was 20 per group. Five fish per group were used to calculate efficiency of colonization. In subsequent time-dependent analysis, three fish were used.

### Determination of LD_50_.

Five different concentrations (1 × 10^5^, 1 × 10^6^, 1 × 10^7^, 1 × 10^8^, and 1 × 10^9^ CFU/mL) of *Sf2a*, *Sb4*, or *SsI* were introduced to 200 mL of autoclaved tap water. Ten fish per group were used to determine the LD_50_ at 30°C and 37°C. Water was changed once after 3 h and then on 21 h followed by every subsequent 24 h for a total of 12 days.

### Preparation of heat-killed immunogen.

Heat-killed (HK) immunogens of *Sf2a*, *Sb4*, and *SsI* strains were prepared and stored as described previously (11). Briefly, overnight grown liquid cultures were spread over tryptic soy agar plates and incubated for 16 h at 37°C. The plates were scraped using a cell scraper the next day and suspended in PBS (pH 7.4). The bacterial cells were then washed, and optical density at 600 nm (OD_600_) was adjusted so that the bacterial concentration became 1 × 10^9^ CFU/mL. The suspensions were heated at 70°C for 1 h under normal pressure. The nonviability was checked, and the immunogens were stored at −80°C for further use.

### Bath immunization.

Three heat-killed bacterial strains were mixed at a ratio of 1:1:1 and adjusted to a total of 3 × 10^9^ CFU/mL. This was added to 50 mL of water. Naive fish were kept in the water for 2 h and then washed with autoclaved tap water and placed in beakers containing 200 mL of autoclaved tap water at 28°C. Prime-boost-boost immunization was carried out with a 14-day interval between two successive doses. Three fish were separately immunized with 1 × 10^9^ CFU/mL of the Δ*hfq* strain.

### Protective efficacy assay.

Immunized fish were challenged with LD_50_ of *Sf2a*, *Sb4*, and *SsI*, separately. Fish were kept at 37°C for 18 h, and intestinal homogenates were plated (*n* = 5). Water was changed once at 3 hpi. Spread in water was also assessed. Efficacy of the live-attenuated Δ*hfq* immunogen was also checked in the same way.

### Survival assay.

Twenty zebrafish per group were kept in autoclaved tap water and challenged with LD_50_ inoculum of *Shigella* strains. Water was changed as stated above until day 12.

### Transmission experiments.

One LD_50_ of bacteria was inoculated in 200 mL autoclaved tap water at 37°C. Fish were divided into four groups, namely, donor-nonimmunized (DNI), recipient-nonimmunized (RNI), donor-immunized (DI), and recipient-immunized (RI) fish. Donor fish, which received the dosage mentioned above, were removed from the water after 3 h, washed in gentamicin (50 μg/mL)-containing autoclaved tap water, and transferred into 200 mL fresh autoclaved tap water with 3 new naive/recipient fish. They were kept at 37°C until 18 hpi. Afterwards, fish (*n* = 3) were dissected, and their intestines were washed, homogenized, serially diluted, and plated. Excretion of *Shigella* in water was also assessed.

### Assay of bacteriolytic activity.

Hearts (as a proxy of blood) of immunized and nonimmunized fish were isolated on the 35th day post 1st immunization. They were homogenized separately in PBS (pH 7.4) along with 2% sodium azide and phenylmethylsulfonyl fluoride (PMSF). Extracts were then incubated with wild-type Shigella flexneri *2a*, Shigella boydii *4*, and Shigella sonnei phase I. Initial inoculum with fresh mid-log-phase bacterial culture was set at 1 × 10^7^ CFU/mL. Thirty microliters of the fish heart extract was incubated with the indicated number of *Shigella* strains separately at 37°C for 1 h. Fresh medium was then added and incubated for another hour, followed by plating.

### Zebrafish as a therapeutic model organism.

A probiotic/commensal mixture was prepared using Lactobacillus acidophilus and Escherichia coli K-12 in a 1:1 ratio (1 × 10^8^ CFU/bacteria/fish). This mixture was used either alone or in combination with a prior antibiotic treatment. The first group received the probiotic mixture alone, the second group received antibiotics (pen-strep 10,000 U/mL), and the third group received an antibiotic treatment followed by probiotic/commensal treatment prior to *Shigella* challenge. Fish were challenged with any one of the three *Shigella* strains at a same dose. Probiotic/commensal treatment or *Shigella* challenge was done using the same protocol mentioned above. For antibiotic treatment, however, fish were kept in antibiotics for 18 h prior to administration. A minimum gap of 4 h was ensured between an antibiotic treatment and the exposure to bacteria. Bacterial colonization was measured at 18 hpi. Fish water was also checked for dissemination of bacteria.

### H&E staining.

Intestinal sections were fixed with 10% buffered formalin and embedded in paraffin. Six-micrometer sections were cut and stained with hematoxylin and eosin (H&E). The stained sections were observed under an Olympus IX51 light microscope. No pathological scores were provided.

### Statistical analyses.

Data were expressed as mean ± standard deviation (SD). Three technical and three biological repeats were carried out, as needed. GraphPad Prism versions 5 and 8.2 were used to carry out all the statistical analyses. Analysis of variance (ANOVA) and one- and two-tailed (for transmission and other cases, respectively) student’s *t* tests were used, as necessary. Tukey’s multiple-comparison test was used to analyze statistically significant differences between the groups. Statistical significance was determined from the *P* values as mentioned in the figure legends.
