# Supplementary material for: An Experimental Adult Zebrafish Model for Shigella Pathogenesis, Transmission, and Vaccine Efficacy Studies
Source: Microbiol Spectr. 2022 May 23;10(3):e00347-22. doi: 10.1128/spectrum.00347-22 (PMC9241715; doi:10.1128/spectrum.00347-22)
Supplement: SUPPLEMENTAL FILE 1 — Supplemental material. Download spectrum.00347-22-s001.pdf, PDF file, 0.2 MB [file spectrum.00347-22-s001.pdf]

Supplemental 1. *LD50 determination*

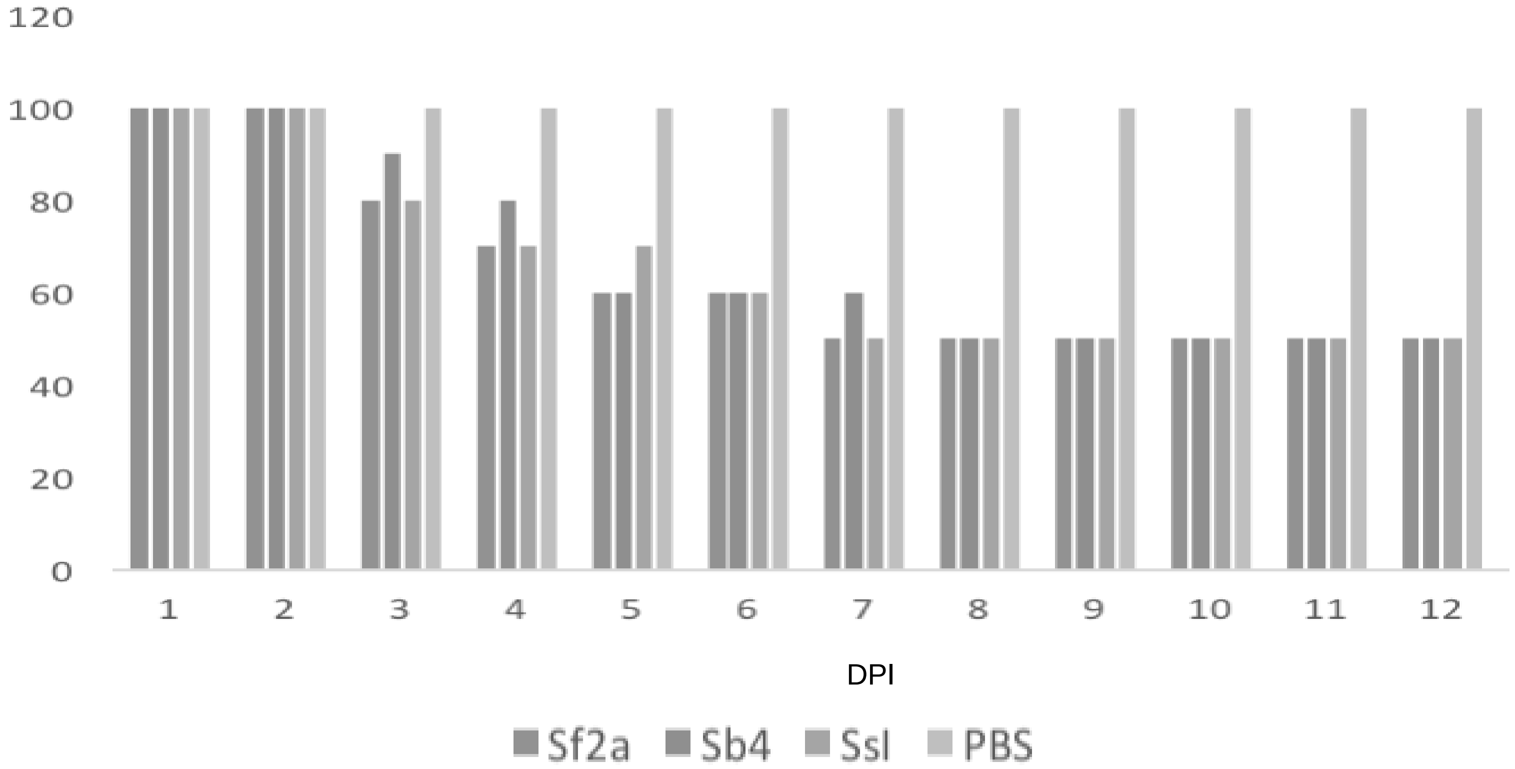

Supplemental 1. Determination of LD50 at 37 degree C of different strains of Shigella sp. 10 fish per group was kept in 200 ml autoclaved tap water and were infected with varying doses of inoculum, as stated in the methods. Since 2 x 10^{7} CFU/ml was observed to be the LD50 value unanimously, only that is being presented here. Water was once changed after 3 hours and then at a 24 hours interval. Fish were kept for 12 days at 37 degree C for observation and their mortality was checked. Data represented here was the result of a single experiment, but the same has been carried out for 5 times before progressing toward any other experiments.

Supplemental 2. **_Colonization of fish gut by LD50 dose in 30 degree C_**

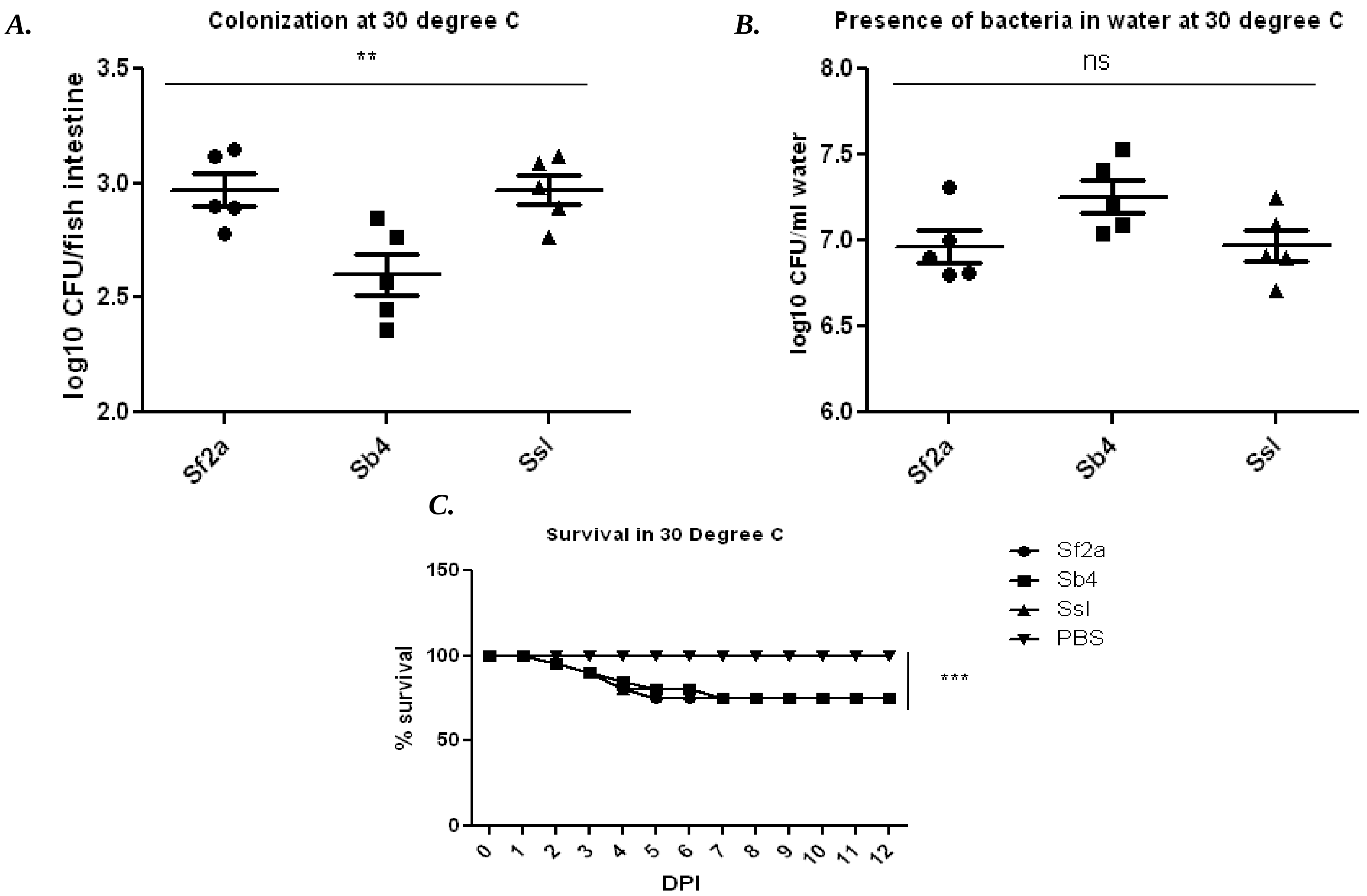

Supplemental 2. Colonization of fish gut at 30 degree C with the pre-determined LD50 dose. Fish were challenged with 2 x 10^{7} CFU/ml bacteria and kept for 24 hours. Water was changed once at 3 hours post infection. 5 fish per group were taken for colonization studies and 10 fish per group were kept for survival studies. ***A.*** Colonization in fish gut and ***B.*** Spread in water was recorded. ***C.*** Survival of the fish was also assessed with the same dose. Much less colonization of the fish gut was observed and fish were surviving the infection. This indicates towards a temperature-dependant infection of *Shigella sp.* in the fish gut. Each dot represent a single fish. Each dot in **B**. represents presence in water. **P<0.05, ***P<0.005

Supplemental 3.

Bath immunization and challenge regimen

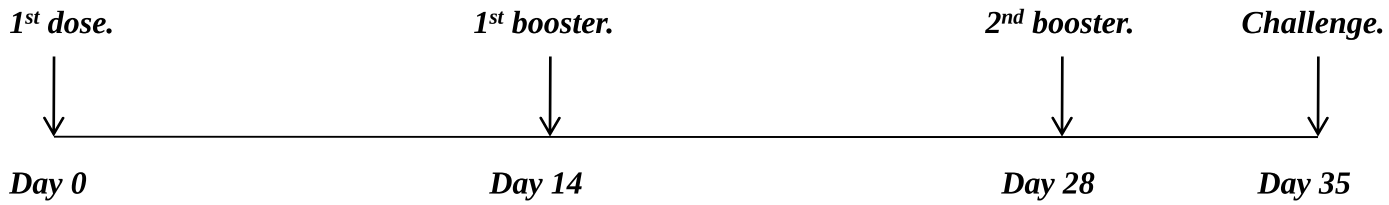

Supplemental 3. Bath immunization and challenge regimen. Fish immunized by bath immunization following the scheme above. Primary immunization was marked by day 0 which was followed by two booster doses on days 14 and 28. Each dose contained 3 x 10^9 CFU/ml heat killed tri-valent *Shigella* immunogen. Fish were challenged on day 35 after first immunization.

Supplemental 4.

Colonization in internal organs

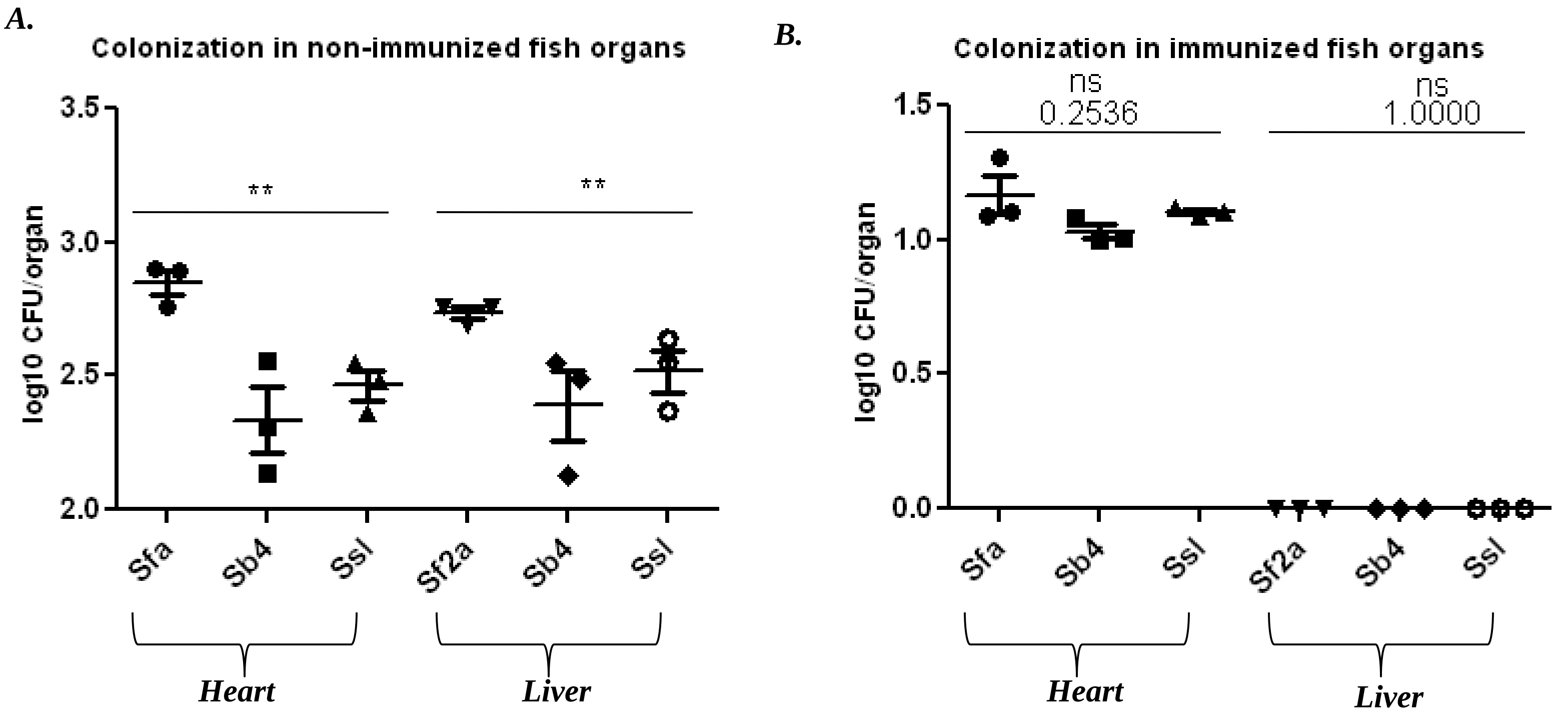

Supplemental 4. Bacterial load in internal organs. Bacterial load in internal organs, such as, heart and liver, was assessed after infecting the fish school with LD50 dose. Heart indicates the systemic spread of the infection via fish blood and liver acts as another site of infection. Both ***A.*** immunized and ***B.*** non-immunized fish were dissected and their organs evaluated. Less and/or no colonization in immunized fish indicates the protective ability of the immunogen used in the study. Each dot represent a single fish organ. **P<0.05.

Bacteriolytic activity

Supplemental 5.

A.

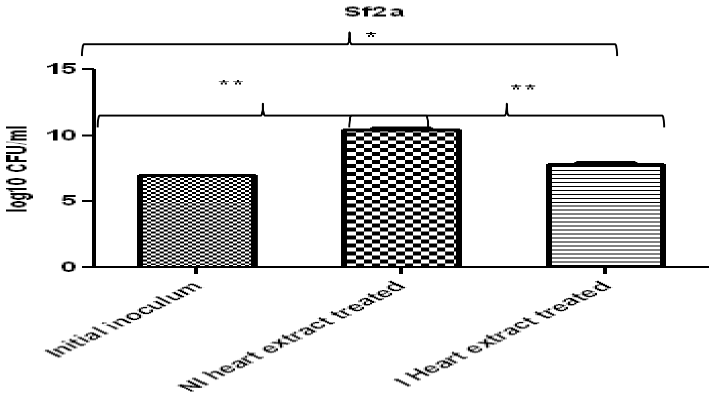

B.

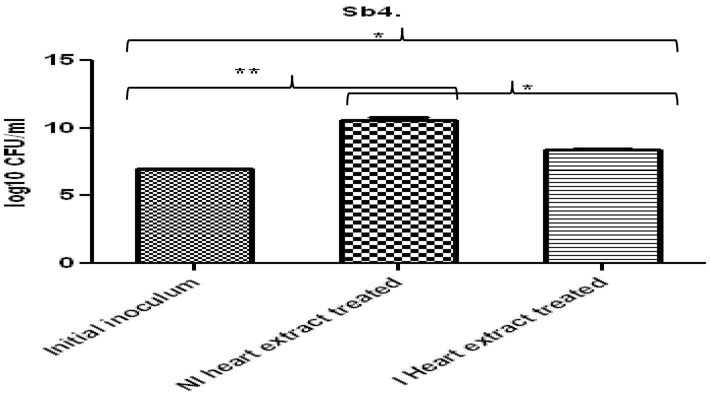

C.

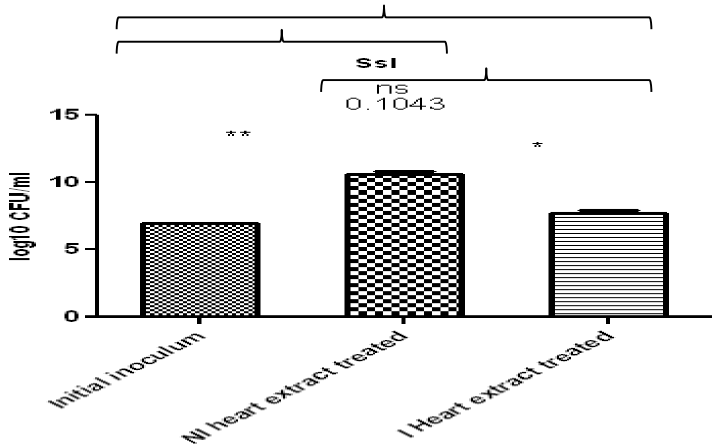

Supplemental 5. Assessment of bacteriolytic activity. Fish were immunized with heat-killed immunogen and their heart (as a proxy of blood) was isolated on day 35th post 1st immunization. Hearts were mashed in PBS (pH 7.4) along with 2% sodium azide and PMSF. The same solution was used to incubate with wild type *Shigella flexneri 2a, Shigella boydii 4* and *Shigella sonnei* phase I. Initial inoculum was set at 1 x 10^7 CFU/ml. Bacteriolytic activity of the fish blood was determined against wild type infections of **A.***Shigella flexneri 2a*, **B.** *Shigella boydii 4*, **C.** *Shigella sonnei* phase I. NI heart – Non-Immunized heart, I heart – Immunized heart. *P<0.05, **P<0.005.

Supplement 6.

Histological analysis

A. **B.** **C.** **D.**

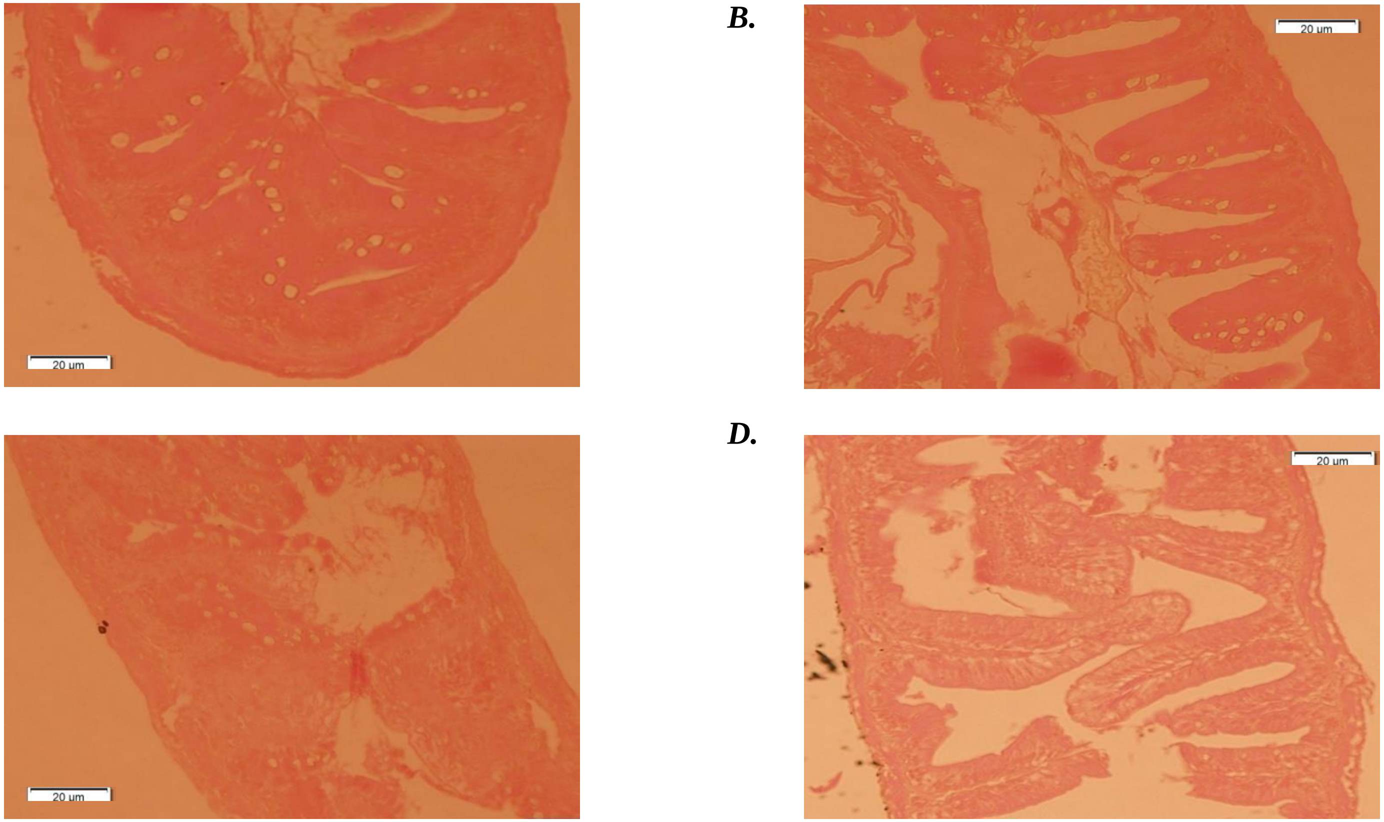

Supplemental 6. Macroscopic signs of lesion and damage to the intestinal villi were found in Shigella infection. Fish were challenged with LD_{50} dose of *Shigella sp.* and observed for macroscopic changes in the gut. ***A.***Control intestine looked without damage and compact in shape. ***B.*** Infection from challenge dose of *Shigella sp.* caused disruption in intestinal villi. ***C.*** Eradication of gut bacteria prior to infection from challenge bacteria caused massive damage to the fish gut. ***D.***Treatment of probiotic, commensal mixture prior to infection from *Shigella sp.* caused the intestinal villi to stay in proper shape and it has caused an increase in intestinal villi.
